# From bench to bedside in endometriosis research: overcoming methodological gaps and enhancing translational potential

**DOI:** 10.1080/07853890.2025.2564913

**Published:** 2025-12-08

**Authors:** Wangshu Li, Jiuxiang Feng, Xiuying Wang

**Affiliations:** Key Laboratory for Early Diagnosis and Biotherapy of Malignant Tumors in Children and Women in Liaoning Province, Dalian Women and Children’s Medical Group, Dalian, Liaoning, China; Dalian Women and Children’s Medical Group Street, No. 154, Zhong Shan Road, Xigang District Dalian, Liaoning Province 116012, China

To the Editor

We read with great interest the recent article entitled *‘1,25(OH)_2_D_3_ alleviates inflammation in endometriosis via VDR-dependent inhibition of the TLR4/NLRP3–NF-κB pathway’* [1]. This work is commendable for systematically linking clinical cell sources, *in vitro* assays, and an *in vivo* rat model to demonstrate that 1,25(OH)_2_D_3_ attenuates LPS-induced inflammation through a VDR-dependent mechanism. Notably, the authors employed the pharmacological antagonist TEI-9647 to confirm the causal involvement of VDR, which strengthens the mechanistic clarity. Such a comprehensive approach provides valuable insights into the immunoendocrine regulation of endometriosis and highlights a potential therapeutic direction with clinical relevance.

Despite these strengths, we believe several limitations deserve further reflection. First, the reliance on pharmacological inhibition alone may not fully prove VDR dependency. TEI-9647, while effective, can exhibit off-target effects. Incorporating genetic approaches, such as VDR knockdown or conditional knockout models, would provide more definitive evidence [2]. Moreover, nuclear translocation of p65 was elegantly shown, but additional assays (e.g. NF-κB reporter activity or chromatin binding) could further validate the functional suppression of NF-κB signaling.

Second, the experimental models only partially mimic the complexity of human endometriosis. The Ishikawa cell line, though widely used, represents adenocarcinoma cells rather than normal endometrial cells. Even with EESCs, the absence of paired eutopic endometrial stromal cells from the same patients limits interpretation of differential responses. *In vivo*, the rat autologous transplantation model lacks menstruation-like cycles, which reduces translational relevance. Future research may benefit from including menstrualized mouse models or patient-derived organoid-immune co-cultures to better replicate the human inflammatory microenvironment [3].

Third, the safety and clinical applicability of 1,25(OH)_2_D_3_ require careful evaluation. While effective in reducing lesion growth and inflammatory markers, supraphysiological dosing carries a risk of hypercalcemia and renal toxicity [4]. The study did not address serum calcium, renal function, or long-term outcomes. Future investigations should integrate safety endpoints, compare active vitamin D with precursor supplementation (e.g. cholecalciferol), and test combination strategies with current hormonal therapies to enhance both efficacy and tolerability.

In conclusion, this article makes a valuable contribution by advancing our understanding of the VDR-mediated anti-inflammatory role of 1,25(OH)_2_D_3_ in endometriosis. By complementing pharmacological evidence with genetic tools, adopting models that more closely resemble the human disease context, and integrating safety evaluations, future studies can bridge the gap from bench to bedside. We appreciate the authors’ important work and look forward to its translation into novel therapeutic approaches for women suffering from endometriosis.
